# Cost-effectiveness of SARS-CoV-2 self-testing at routine gatherings to minimize community-level infections in lower-middle income countries: A mathematical modeling study

**DOI:** 10.1371/journal.pone.0311198

**Published:** 2024-10-04

**Authors:** Megan A. Hansen, Alvin X. Han, Joshua M. Chevalier, Ethan Klock, Hiromi Pandithakoralage, Alexandra de Nooy, Tom Ockhuisen, Sarah J. Girdwood, Nkgomeleng A. Lekodeba, Shaukat Khan, Helen E. Jenkins, Cheryl C. Johnson, Jilian A. Sacks, Colin A. Russell, Brooke E. Nichols

**Affiliations:** 1 Department of Global Health, Amsterdam Institute for Global Health and Development, Amsterdam UMC, University of Amsterdam, Amsterdam, the Netherlands; 2 Department of Global Health, Boston University School of Public Health, Boston, MA, United States of America; 3 Department of Medical Microbiology and Infection Prevention, Amsterdam UMC, University of Amsterdam, Amsterdam, the Netherlands; 4 Health Economics and Epidemiology Research Office, University of the Witwatersrand, Johannesburg, South Africa; 5 FIND, Geneva, Switzerland; 6 Department of Biostatistics, Boston University School of Public Health, Boston, MA, United States of America; 7 World Health Organization (WHO), Geneva, Switzerland; King Khalid University, EGYPT

## Abstract

Places of worship serve as a venue for both mass and routine gathering around the world, and therefore are associated with risk of large-scale SARS-CoV-2 transmission. However, such routine gatherings also offer an opportunity to distribute self-tests to members of the community to potentially help mitigate transmission and reduce broader community spread of SARS-CoV-2. Over the past four years, self-testing strategies have been an impactful tool for countries’ response to the COVID-19 pandemic, especially early on to mitigate the spread when vaccination and treatment options were limited. We used an agent-based mathematical model to estimate the impact of various strategies of symptomatic and asymptomatic self-testing for a fixed percentage of weekly routine gatherings at places of worship on community transmission of SARS-CoV-2 in Brazil, Georgia, and Zambia. Testing strategies assessed included weekly and bi-weekly self-testing across varying levels of vaccine effectiveness, vaccine coverage, and reproductive numbers to simulate developing stages of the COVID-19 pandemic. Self-testing symptomatic people attending routine gatherings can cost-effectively reduce the spread of SARS-CoV-2 within places of worship and the community, resulting in incremental cost-effectiveness ratios of $69-$303 USD. This trend is especially true in contexts where population level attendance at such gatherings is high, demonstrating that a distribution approach is more impactful when a greater proportion of the population is reached. Asymptomatic self-testing of attendees at 100% of places of worship in a country results in the greatest percent of infections averted and is consistently cost-effective but remains costly. Budgetary needs for asymptomatic testing are expensive and likely unaffordable for lower-middle income countries (520-1550x greater than that of symptomatic testing alone), promoting that strategies to strengthen symptomatic testing should remain a higher priority.

## Introduction

Despite no longer being a public health emergency of international concern as of May 2023, COVID-19 continues to take an outsized toll on human life, wellbeing, and the global economy with over 775 million confirmed cases and 7 million deaths reported globally as of June 2024 [1, 2]. Diagnostic testing has been acknowledged as a critical tool to combat the pandemic; however, access to testing remains varied with low income countries (LICs) and lower-middle income countries (LMICs) testing at a fraction of the rate of high income countries (HICs) nearly four years into the pandemic [3].

Antigen-detection rapid diagnostic tests (Ag-RDTs) have lower sensitivity than gold standard diagnostics–reverse transcriptase polymerase chain reaction (RT-PCR) tests. Unlike PCR tests, Ag-RDTs are simple and provide rapid same day results, bringing testing services to settings where laboratories or highly skilled personnel are limited. Using Ag-RDTs for COVID-19 detection is one mechanism to relieve human resource constraints through self-administration and rapidly expand testing capacity. For these reasons, the World Health Organization (WHO) has endorsed the use of Ag-RDTs as self-tests to support COVID-19 management since July 2022 [4]. Self-testing for SARS-CoV-2 became increasingly popular throughout the pandemic, particularly in HIC settings [4]. However, access to and adoption of self-testing varies substantially by region and country income level [3].

Self-testing has served as a valuable tool for tool for several infectious diseases, such as the human immunodeficiency virus (HIV) and Hepatitis C virus (HCV) [5, 6]. It has previously been demonstrated that self-testing is an acceptable, feasible, and cost-effective method at increasing HIV testing uptake in South Africa [5]. HCV self-testing in high prevalence settings in China, Georgia, Vietnam, and Kenya cost-effectively identified people who had never been tested before [6]. The cost-effectiveness of self-testing for HIV and HCV suggests that self-testing for SARS-CoV-2 could also be a cost-effective method for identifying disease, initiating isolation and/or treatment, and preventing further transmission of the respiratory virus.

Early in the COVID-19 pandemic, it became evident that many SARS-CoV-2 infections occurred at events with large levels of attendance, i.e., mass gatherings [7]. Previous research has analyzed SARS-CoV-2 transmission dynamics of mass gatherings that take place indoors, such as concerts and sports games [8]. These studies have concluded that transmission can be significantly reduced if the proper precautions, such as testing and isolation, are taken prior to the event as opposed to no interventions [9]. Despite the progress in researching SARS-CoV-2 transmission at singular one-time gathering events, a gap in understanding transmission at gatherings occurring at regularly scheduled time intervals remains [10]. Gatherings at places of worship typically occur at routine time intervals and are highly attended in many LMICs [11]. Gatherings at places of worship serve as an opportunity to enable wide-scale and routine access to self-testing among a significant proportion of the community [11]. These characteristics make places of worship a possible venue to not only mitigate transmission within the mass gathering event itself, but to also reduce community spread of SARS-CoV-2 more broadly.

The purpose of this study was to investigate the impact and cost-effectiveness of COVID-19 self-testing prior to routine gatherings–such as in places of worship, using an agent-based mathematical model in three LMICs: Brazil, Georgia, and Zambia. We aimed to understand how different self-testing strategies impact the dynamics of SARS-CoV-2 transmission at the routine gatherings and within the surrounding community. Finally, we assessed which self-testing strategies were the most impactful, the most cost-effective, and the most affordable. At this time in the pandemic, understanding the impact of distribution and self-testing within different routine contexts remains a priority as countries develop more sustainable COVID-19 management strategies. This analysis provides evidence on how to implement routine testing strategies in a useful and cost-effective manner.

## Materials and methods

We utilized the results from an agent-based model, Propelling Action for Testing and Treatment (PATAT), to analyze the impact and cost-effectiveness of different strategies of self-testing for SARS-CoV-2 at places of worship in LMICs [12–14]. PATAT is a stochastic agent-based model that was parameterized to various country archetypes and informed by population demographic information, urban/rural geography, healthcare structure, work structure, and school structure. To understand transmission dynamics at places of worship, we parameterized PATAT to three different demographic contexts and simulated five different testing strategies with 24 combinations of varying epidemic conditions, resulting in 217 scenarios per country that were run five times each. The results from each of the five runs for the 217 scenarios were then averaged together, and the standard deviation was calculated to produce a robust set of results. A cost-effectiveness analysis was performed to calculate the incremental cost-effectiveness ratios (ICERs) of each scenario. The modeling results were exported from Python, statistical analyses were conducted in Microsoft Excel (Version 16.65), and the cost-effectiveness analysis and figure generation were performed in R Studio (Version 2022.07.2).

### Study population

To understand transmission dynamics at routine gatherings within places of worship, we parameterized PATAT to three different demographic contexts: Brazil, Georgia, and Zambia [15–17]. The parameters used to characterize the average routine gathering size at a place of worship and the proportion of the population attending places of worship for each country are summarized in Table 1 [18–20]. Additional country specific demographic characteristics used in PATAT are outlined in S1 Table.

**Table 1:** Routine gathering attendee and population-level parameters.

Parameter	Brazil [15, 18]	Georgia [16, 19]	Zambia [17, 20]
Total population size	212.6 M	3.99 M	18.38 M
Average household size	3.3	3.3	5.0
Average routine gathering size (standard deviation)	200 (+100)	200 (+100)	500 (+100)
Proportion of population attending places of worship	41.1%	13.0%	70.0%
Average random contacts in routine gathering per person	10*	10*	10*

*Assumption

The size of a religious gathering is assumed to follow a normal distribution of the country’s mean and standard deviation described in Table 1. PATAT assumes that, of a proportion of the population attending places of worship, all members of a household will visit the religious gathering every Sunday. In addition to being in close contact with each other, each household member also has a random number of close contacts from other households that attend the same religious gathering drawn from a Gamma distribution. Places of worship are ordered such that proximally ordered households in the same neighborhood visit the same religious gathering [13].

### Epidemic conditions

Table 2 shows the different epidemic conditions considered for the scenarios simulated in PATAT. Scenarios were further stratified by vaccine effectiveness, vaccine coverage, and reproductive numbers (Rt) to simulate varying stages of the COVID-19 pandemic. Additional SARS-CoV-2 transmission related parameters, testing parameters, and isolation and quarantine parameters used in the PATAT model are respectively outlined in S2–S4 Tables.

**Table 2:** Epidemic condition parameters.

Parameter	Values
Effective reproductive number (Rt)	0.9, 1.2. 1.5, 2.0
Vaccine coverage*	10%, 50%, 80%
Vaccine effectiveness	High (70% protective against severe disease), Low (30% protective against severe disease)

*For individuals of at least 18 years of age

### Modeling scenarios

Using the results from PATAT, we were able to estimate how various strategies of asymptomatic self-testing of a fixed percentage of persons attending routine gatherings at places of worship–in addition to the general underlying level of symptomatic testing in the population–would impact both transmission at the place of worship as well as overall community transmission. The underlying population-level symptomatic COVID-19 testing rate was parameterized to each country as of June 2022–30 tests per 100,000 people per day in Brazil, 50 tests per 100,000 people per day in Georgia, and 10 tests per 100,000 per day in Zambia [3]. It was assumed that after a positive test, 50% would enter self-isolation, of which 86% would complete a full week (7 days) [12].

After first simulating the baseline for each respective country–or background population-level symptomatic testing–we simulated the following potential testing strategies: 1) symptomatic testing amongst those attending places of worship; 2) symptomatic testing at all places of worship + asymptomatic self-testing of 10% of places of worship nationwide; 3) symptomatic testing at all places of worship + asymptomatic self-testing of 20% of places of worship nationwide; 4) symptomatic testing at all places of worship + asymptomatic self-testing of 40% of places of worship nationwide; 5) symptomatic testing at all places of worship + asymptomatic self-testing of 100% of places of worship nationwide. For scenarios 2–5, both weekly testing and bi-weekly testing were assessed.

To better understand the impact that each self-testing strategy had on reducing SARS-CoV-2 transmission, we simulated results for both infections that occurred in the community and infections that were related to attendance at places of worship. These model outputs included: total infections, total infections averted, percent of infections averted, and number of tests used per infection averted (measure of testing efficiency), and the number of deaths.

### Cost-effectiveness analysis

Each of the self-testing strategies were analyzed for their incremental cost-effectiveness in an economic evaluation with a total cost per self-test of $2.50 USD (2023) [21, 22]. This cost includes a purchasing cost of $1.00 USD per test kit, comprising of 40% of the total distribution cost of self-tests from the provider perspective [22, 23]. The cost $2.50 test considers the costs for several economic factors such as: test procurement, test distribution, test storage, staff overhead (training, communication, management, service delivery), data management, and necessary consumables [22]. The total number of infections related to attendance at routine gatherings averted was used as the effect measure in the analysis. We determined the total cost of self-test provision per infection averted, as well as the ICER for each scenario, under every epidemic condition in all three country contexts. The Consolidated Health Economic Evaluation Reporting Standards (CHEERS) were followed and are reported in S5 Table [24].

### Ethics

This research utilizes a stochastic agent-based model that was parameterized using publicly available data from the literature. Given the nature of the study, formal ethical approval was not required. However, ethical considerations were included throughout the research process. Transparency was maintained in the reporting of our modeling methodology, allowing for scrutiny and replication, while our raw results of the model output are available in S1 File. We declare no conflicts of interest that could influence the research process or outcomes. This statement affirms our commitment to ethical conduct in research, even in the absence of formal approval.

## Results and discussion

**Fig 1:**
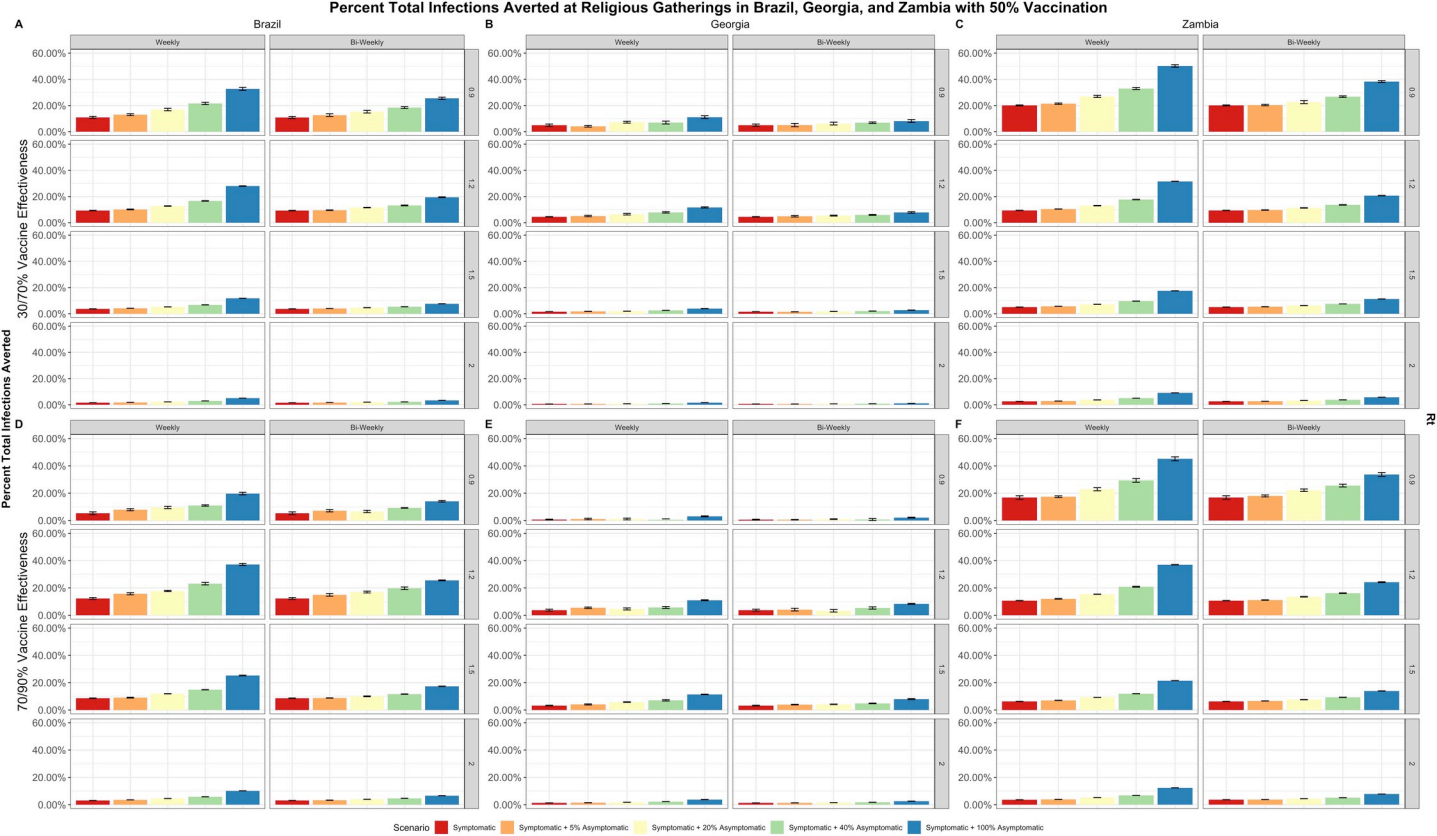
Percent of total community infections averted. Percent of total community infections averted over a 90-day period with vaccination coverage levels of 50%, varied by Rt (right), frequency of testing (top), and vaccine effectiveness (left). The five different testing scenarios are represented by the different colors in the figure: 1. Symptomatic testing (red), 2. Symptomatic and 5% asymptomatic testing (orange), 3. Symptomatic and 20% asymptomatic testing (yellow), 4. Symptomatic and 40% asymptomatic testing (green), 5. Symptomatic and 100% asymptomatic testing (blue). Panels A and D display outcomes in Brazil, panels B and E display outcomes in Georgia, and panels C and F display outcomes in Zambia [25–27].

**Fig 2:**
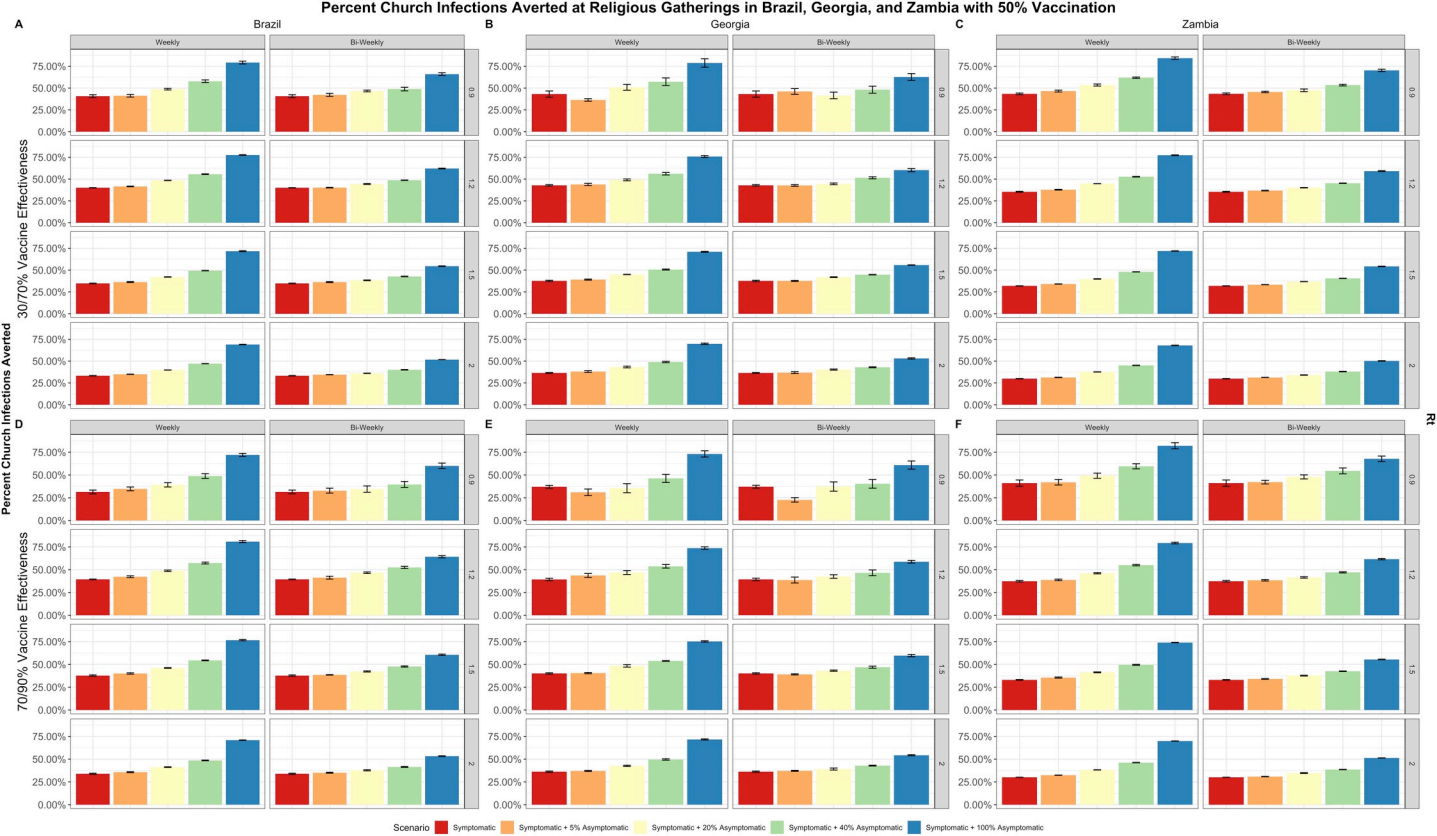
Percent of place of worship infections averted. Percent of infections averted within places of worship over a 90-day period with vaccination coverage levels of 50%, varied by Rt (right), frequency of testing (top), and vaccine effectiveness (left). The five different testing scenarios are represented by the different colors in the figure: 1. Symptomatic testing (red), 2. Symptomatic and 5% asymptomatic testing (orange), 3. Symptomatic and 20% asymptomatic testing (yellow), 4. Symptomatic and 40% asymptomatic testing (green), 5. Symptomatic and 100% asymptomatic testing (blue). Panels A and D display outcomes in Brazil, panels B and E display outcomes in Georgia, and panels C and F display outcomes in Zambia [25–27].

Despite differences in the proportion of populations regularly attending places of worship in the three countries, all testing strategies are found to be most effective at averting infections within the overall community under moderate epidemic conditions–either when the Rt is low and population immunity (quantified as vaccine effectiveness) is moderate, or when the Rt is high and population immunity is high (Fig 1). The same also holds true for averting infections within routine gatherings at places of worship (Fig 2). These results at moderate epidemic conditions suggest that implementing an adaptable testing program necessitates reliable underlying community surveillance to identify when an epidemic wave may be approaching, allowing self-testing in strategic settings, such as places of worship, to be scaled accordingly [25–27]. Community surveillance of COVID-19 cases may be especially important for LMICs where resources for diagnostics and treatment options may be limited. Such robust systems do not currently exist in resource limited settings; therefore, surveillance for preparedness purposes for COVID-19 or other emerging infectious diseases should be strengthened to identify when there are shifts in the epidemic (e.g., due to emerging variants) to trigger impactful deployment of self-testing in strategic settings.

Testing all symptomatic persons (either weekly or biweekly), can cost-effectively avert 2%–16% of total community infections in Brazil, and 31%–45% of infections occurring at routine gatherings at places of worship in Brazil, varying according to all epidemic conditions modeled. Similarly, in Georgia, 1%–6% of total community infections and 28%–45% routine gathering attendance-related infections can be averted with weekly and biweekly screening of symptomatic persons; in Zambia, 2%–21% of total infections and 29%–45% of routine gathering attendance-related infections (Table 3). Under all epidemic conditions modeled: testing asymptomatic persons in addition to existing symptomatic testing can avert 3%–34% of community infections and 52%–82% of infections at routine gatherings in Brazil, 1%–10% of community infections and 53%–80% of infections at routine gatherings in Georgia, and 5%–52% of community infections and 49%–85% of infections at routine gatherings in Zambia (Table 3).

**Table 3:** Cost-effectiveness analysis of differing testing strategies by country.

Scenario	Percent community infections averted (%)	Incremental cost per community infection prevented (USD $)	Percent place of worship infections averted (%)	Incremental cost per infection prevented within routine gatherings / places of worship (USD $)	Total national cost (USD $) over a 90-day period
**Brazil**					
**Symptomatic testing only**	2–16	3–42	31–45	74–175	$3.8 million–$96.7 million
**Weekly asymptomatic self-testing 100%**	3–34	161–11,838	52–82	2889–57,457	$1.6 billion–$2.9 billion
**Georgia**					
**Symptomatic testing only**	1–6	3–37	28–46	85–303	$987,500 –$575,000
**Weekly asymptomatic self-testing 100%**	1–10	165–18,693	53–80	2,995–470,245	$8.5 million–$15.6 million
**Zambia**					
**Symptomatic testing only**	2–21	3–38	29–45	69–119	$818,100 –$13.5 million
**Weekly asymptomatic self-testing 100%**	5–52	202–2,020	49–85	2,854–101,580	$232 million–$427 million

Accompanying asymptomatic testing with symptomatic self-testing prevents additional infections but generates slight impact per test for a significantly large increase in cost. Table 3 displays ICERs of the testing strategies that consistently showed cost-effectiveness across the range of epidemic scenarios in Brazil, Georgia, and Zambia. In all three countries, asymptomatic self-testing of 100% of routine gatherings at places of worship in a country results in the greatest percent of infections averted and consistently lands along the cost-effectiveness frontier. The resulting cost of testing 100% of asymptomatic people ranges from $1.6 billion–$2.9 billion USD in Brazil, $8.5 million–$15.6 million USD in Georgia, and $232 million–$427 million USD in Zambia over a 90-day period. This represents a budget increase that is 520–1550 times greater than that of symptomatic testing along for a minor reduction in SARS-CoV-2 transmission. While implementation at 100% of places of worship in a country may not be fully feasible, and comes with hefty upfront costs, costing studies have shown that the cost per self-test distributed can be expected to decrease as self-testing becomes a routine practice–overhead costs become negligent, and test procurement costs decrease with greater access and availability [22]. The exact willingness to pay and invest in routine self-testing infrastructure will differ vastly between countries and will also depend on competing health priorities and availability of funding; for example, where HICs spent an average of 14% of Gross Domestic Product (GDP) in 2020 on COVID-19 related health interventions, LICs spent on average only 1% of GDP [28].

Self-testing in places of worship has the greatest impact on reducing SARS-CoV-2 infections that occur within the places of worship themselves; the percentage of infections averted at the community level are directly proportional to the percentage of the population attending such routine gatherings (Fig 1). For example, in Brazil and Zambia, where 41% and 70% of people regularly attend places of worship, there is a larger percentage of infections averted compared to Georgia, where only 13% of the population routinely attend services at places of worship (Table 1). This analysis uses routine gatherings at places of worship as a surrogate for the scale-up of community-based testing; in contexts where country-level attendance in places of worship is lower, there may be alternative routine community settings to reach increased proportions of the population to offer self-testing and ensure greater impact. We recognize that gatherings at places of worship are not the only occasion in which routine gatherings take place–our results can be applied to any community gathering that is regularly scheduled whether it is cultural, educational, recreational, professional, or social. In addition to distribution of self-tests at these gatherings, it may also be useful to utilize this opportunity as a means to administer other public health intervention tools, such as health communication and vaccination services.

Our modeling analysis comes with several important limitations. Firstly, the model was parameterized to the BA.1 Omicron variant, so it may not reflect the behavior of future variants with respect to their transmissibility, vaccine effectiveness, or underlying immunity almost four years into the pandemic. Additionally, it was assumed that vaccination is only available for individuals 18 years of age and older despite many countries having authorized COVID-19 vaccination for children, implying we may potentially be underestimating the effect of vaccination among the modeled population [29]. To address these limitations, we varied vaccination coverage (from 10% to 80%) and vaccine effectiveness (from 30% to 70%) to increase the robustness of our model around these measures. Secondly, we did not consider other mitigation strategies, such as masking or social distancing. However, these measures have been widely discontinued and the re-implementation of such strategies of to prevent transmission is unlikely at the current state of SARS-CoV-2 circulation. Thirdly, we assumed the total cost to offer a COVID-19 self-test would be USD 2.50 from estimates in the literature, but depending on the purchasing cost or distribution modality, this could vary by country. Also, it is possible that this cost could be covered by country governments, healthcare systems, or private implementers. While varying the purchasing and administration cost of the test changes the magnitude of the ICER, it does not change the magnitude of difference between scenarios. Therefore, our conclusions are unlikely to change when assuming a different cost of a self-test.

Finally, and most notably, our model uses routine gatherings at places of worship as a proxy for other routine gatherings as mentioned before; therefore, we recognize it may be beneficial for future modeling analyses to parameterize models to other routine gathering contexts that happen at different scales and frequencies. However, additional COVID-19 self-testing modeling studies have evaluated such settings (schools and workplaces), and similarly observe that prioritizing the self-testing of symptomatic people for asymptomatic is cost-effective and the most appropriate use of resources [29]. Given the robustness of these results and shared conclusions among different settings, changing the type and frequency of routine gatherings is unlikely to change our primary finding that symptomatic testing is the most cost-effective use of available budgets. Therefore, these results can also be used as evidence to provide guidance for the strategic implementation of self-testing of large groups of people at routine time intervals for other infectious diseases, such as respiratory viruses that may share similar epidemiological characteristics, or potential future pandemics.

While there have been previous studies, both modeled and empiric, that examine the spread of SARS-CoV-2 at routine gatherings (i.e., concerts and sports games), this paper is the first to our knowledge to aim to understand how routine self-testing at regularly scheduled events (i.e., religious gatherings) might reduce COVID-19 transmission both within the event itself and the broader community [30]. Additionally, this is the first paper to perform an economic analysis of several COVID-19 self-testing strategies at places of worship and routine gathering events, meanwhile demonstrating which frequencies of testing are the most cost-effective in a resource limited setting [31]. Our results are generalizable to other LMICs, as our analysis examines three countries based in different continents, each of varying income-levels and population demographics, while producing consistent results on the cost-effectiveness of symptomatic self-testing for SARS-CoV-2.

## Conclusions

Though the COVID-19 pandemic is no longer considered an international emergency, SARS-CoV-2 continues to circulate, and countries are now in a position to set adaptable strategies to mitigate the ongoing threat posed by the virus–including how best to consider the use of routine self-testing. Ultimately, targeting routine gatherings at places of worship as a mechanism to distribute tests to community members can effectively reduce community-level SARS-CoV-2 transmission but comes at substantial cost that is likely not feasible or affordable in LMIC settings. Testing symptomatic persons routinely attending places of worship can significantly reduce community spread of SARS-CoV-2 in populations where attendance at such events is high and can be cost-effective. Accordingly, to effectively target self-testing approaches as part of pandemic response and preparedness, programs need to prioritize health communication in settings where large proportions of the population can be reached, which may include routine gatherings in places of worship, while also ensuring the introduction or strengthening of sufficient community surveillance. Lessons from this study suggest engaging with places of worship, and other community-based routine gatherings, can be a strategic way to offer testing in a cost-effective manner in future pandemics.

## Supporting information

S1 Table Country specific demographic characteristics used in the PATAT model. (PDF)

S2 Table SARS-CoV-2 transmission related parameters used in the PATAT model. (PDF)

S3 Table. Testing parameters used in the PATAT model (PDF)

S4 Table Isolation and quarantine parameters used in the PATAT model. (PDF)

S5 Table CHEERS checklist. (PDF)

S1 File Raw results of model output. (XLSX)

## References

[1] Statement on the fifteenth meeting of the IHR (2005) Emergency Committee on the COVID-19 pandemic [Internet]. [cited 2024 Jun 28]. Available from: https://www.who.int/news/item/05-05-2023-statement-on-the-fifteenth-meeting-of-the-international-health-regulations-(2005)-emergency-committee-regarding-the-coronavirus-disease-(covid-19)-pandemic

[2] COVID-19 cases | WHO COVID-19 dashboard [Internet]. [cited 2024 Jun 28]. Available from: https://data.who.int/dashboards/covid19/cases

[3] FIND COVID-19 Test tracker [Internet]. [cited 2024 Jun 28]. Available from: https://www.finddx.org/tools-and-resources/dxconnect/test-directories/covid-19-test-tracker/

[4] WHO issues its first emergency use listing for a SARS-CoV-2 self-test | WHO—Prequalification of Medical Products (IVDs, Medicines, Vaccines and Immunization Devices, Vector Control) [Internet]. [cited 2024 Jun 28]. Available from: https://extranet.who.int/prequal/news/who-issues-its-first-emergency-use-listing-sars-cov-2-self-test

[5] Jamieson L, Johnson LF, Matsimela K, Sande LA, d’Elbée M, Majam M, et al. The cost effectiveness and optimal configuration of HIV self-test distribution in South Africa: a model analysis. BMJ Global Health. 2021 Jul 1;6(Suppl 4):e005598. doi: 10.1136/bmjgh-2021-005598 34275876 PMC8287627

[6] Walker JG, Easterbrook P, Fajardo E, Ivanova E, Jamil MS, Johnson C, et al. Cost-effectiveness of hepatitis C virus self-testing. In: Recommendations and guidance on hepatitis C virus self-testing [Internet]. World Health Organization; 2021 [cited 2024 Jun 28]. Available from: https://www.ncbi.nlm.nih.gov/books/NBK572736/

[7] Key planning recommendations for mass gatherings in the context of COVID-19 [Internet]. [cited 2024 Jun 28]. Available from: https://www.who.int/publications/i/item/10665-332235

[8] Yasutaka T, Murakami M, Iwasaki Y, Naito W, Onishi M, Fujita T, et al. Assessment of COVID-19 risk and prevention effectiveness among spectators of mass gathering events. Microbial Risk Analysis. 2022 Aug 1;21:100215. doi: 10.1016/j.mran.2022.100215 35382415 PMC8969296

[9] Revollo B, Blanco I, Soler P, Toro J, Izquierdo-Useros N, Puig J, et al. Same-day SARS-CoV-2 antigen test screening in an indoor mass-gathering live music event: a randomised controlled trial. The Lancet Infectious Diseases. 2021 Oct 1;21(10):1365–72. doi: 10.1016/S1473-3099(21)00268-1 34051886 PMC8457773

[10] Estelle CD, Perl TM. To Test or Not to Test: COVID-19 Prevention Strategies to Keep Large Gatherings Safe. Ann Intern Med. 2021 Oct 19;174(10):1470–1. doi: 10.7326/M21-2976 34280334 PMC8296719

[11] Hoang VT, Gautret P, Memish ZA, Al-Tawfiq JA. Hajj and Umrah Mass Gatherings and COVID-19 Infection. Curr Trop Med Rep. 2020 Nov 3;7(4):133–40. 33169095 10.1007/s40475-020-00218-x PMC7609349

[12] Han AX, Girdwood SJ, Khan S, Sacks JA, Toporowski A, Huq N, et al. Strategies for Using Antigen Rapid Diagnostic Tests to Reduce Transmission of Severe Acute Respiratory Syndrome Coronavirus 2 in Low- and Middle-Income Countries: A Mathematical Modelling Study Applied to Zambia. Clinical Infectious Diseases. 2023 Feb 15;76(4):620–30. doi: 10.1093/cid/ciac814 36208211 PMC9619661

[13] Han AX, Hannay E, Carmona S, Rodriguez B, Nichols BE, Russell CA. Estimating the potential impact and diagnostic requirements for SARS-CoV-2 test-and-treat programs. Nat Commun. 2023 Dec 2;14(1):7981. doi: 10.1038/s41467-023-43769-z 38042923 PMC10693634

[14] Han AX, Toporowski A, Sacks JA, Perkins MD, Briand S, van Kerkhove M, et al. SARS-CoV-2 diagnostic testing rates determine the sensitivity of genomic surveillance programs. Nat Genet. 2023 Jan 9;55(1):26–33. doi: 10.1038/s41588-022-01267-w 36624344 PMC9839449

[15] Zambia Statistics Agency–Quality Statistics for Development [Internet]. 2024 [cited 2024 Jul 16]. Available from: https://www.zamstats.gov.zm/

[16] Brazilian Institute of Geography and Statistics, IBGE–Censo 2010 [Internet]. [cited 2024 Jul 16]. Available from: https://censo2010.ibge.gov.br/

[17] National Statistics Office of Georgia–Census, 2014 –მთავარი [Internet]. [cited 2024 Jul 17]. Available from: https://www.geostat.ge/en

[18] The Age Gap in Religion Around the World [Internet]. Pew Research Center. 2018 [cited 2024 Jul 16]. Available from: https://www.pewresearch.org/religion/2018/06/13/the-age-gap-in-religion-around-the-world/

[19] NDI time-series dataset Georgia [Internet]. Caucas Research Resource Center. 2020 [cited 2024 Jul 16]. Available from: https://www.caucasusbarometer.org/en/ndi-ge/RELSERV/

[20] Report on International Religious Freedom: Zambia [Internet]. United States Department of State. [cited 2024 Jul 16]. Available from: https://www.state.gov/reports/2020-report-on-international-religious-freedom/zambia/

[21] Support Package including US $7 million investment accelerates availability of affordable COVID-19 self-tests in low- and middle-income countries [Internet]. FIND. [cited 2024 Jul 16]. Available from: https://www.finddx.org/publications-and-statements/support-package-including-us7-million-investment-accelerates-availability-of-affordable-covid-19-self-tests-in-low-and-middle-income-countries/

[22] Hansen MA, Lekodeba NA, Chevalier JM, Ockhuisen T, Rey-Puech P del, Marban-Castro E, et al. Cost of SARS-CoV-2 self-test distribution programmes by different modalities: a micro-costing study in five countries (Brazil, Georgia, Malaysia, Ethiopia and the Philippines). BMJ Open. 2024 Apr 1;14(4):e078852. doi: 10.1136/bmjopen-2023-078852 38631825 PMC11029185

[23] Sande LA, Matsimela K, Mwenge L, Mangenah C, Choko AT, d’Elbée M, et al. Costs of integrating HIV self-testing in public health facilities in Malawi, South Africa, Zambia and Zimbabwe. BMJ Global Health. 2021 Jul 1;6(Suppl 4):e005191. 10.1136/bmjgh-2021-005191 PMC8287606 34275874

[24] Husereau D, Drummond M, Augustovski F, de Bekker-Grob E, Briggs AH, Carswell C, et al. Consolidated Health Economic Evaluation Reporting Standards (CHEERS) 2022 Explanation and Elaboration: A Report of the ISPOR CHEERS II Good Practices Task Force. Value Health. 2022 Jan;25(1):10–31. doi: 10.1016/j.jval.2021.10.008 35031088

[25] COVID-19 Results Briefing: Brazil [Internet]. Institute for Health Metrics and Evaluation, IHME. [cited October 13, 2022]. Available from: https://www.healthdata.org/research-analysis/diseases-injuries/covid/policy-briefings

[26] COVID-19 Results Briefing: Georgia [Internet]. Institute for Health Metrics and Evaluation, IHME. [cited October 13, 2022]. Available from: https://www.healthdata.org/research-analysis/diseases-injuries/covid/policy-briefings

[27] COVID-19 Results Briefing: Zambia [Internet]. Institute for Health Metrics and Evaluation, IHME. [cited October 13, 2022]. Available from: https://www.healthdata.org/research-analysis/diseases-injuries/covid/policy-briefings

[28] González López-Valcárcel B, Vallejo-Torres L. The costs of COVID-19 and the cost-effectiveness of testing. Applied Economic Analysis. 2021 Jan 1;29(85):77–89.

[29] Chevalier JM, Han AX, Hansen MA, Klock E, Pandithakoralage H, Ockhuisen T, et al. Impact and cost-effectiveness of SARS-CoV-2 self-testing strategies in schools: a multicountry modelling analysis. BMJ Open. 2024 Feb 1;14(2):e078674. doi: 10.1136/bmjopen-2023-078674 38417953 PMC10900377

[30] Aziz NA, Othman J, Lugova H, Suleiman A. Malaysia’s approach in handling COVID-19 onslaught: Report on the Movement Control Order (MCO) and targeted screening to reduce community infection rate and impact on public health and economy. Journal of Infection and Public Health. 2020 Dec 1;13(12):1823–9. doi: 10.1016/j.jiph.2020.08.007 32896496 PMC7456294

[31] Quadri SA. COVID-19 and religious congregations: Implications for spread of novel pathogens. International Journal of Infectious Diseases. 2020 Jul 1;96:219–21. doi: 10.1016/j.ijid.2020.05.007 32389851 PMC7204705

