# Supplementary material for: Cost-effectiveness of SARS-CoV-2 self-testing at routine gatherings to minimize community-level infections in lower-middle income countries: A mathematical modeling study
Source: PLoS One. 2024 Oct 4;19(10):e0311198. doi: 10.1371/journal.pone.0311198 (PMC11451991; doi:10.1371/journal.pone.0311198)
Supplement: S1 Table — (PDF) [file pone.0311198.s001.pdf]

S1 Table. Country specific demographic characteristics used in the PATAT model [1].

**Parameter**	**Values/Distribution**	**Reference**
	Population demography	
Total simulation population	1,000,000	
Mean household size	Brazil: 3.3<br>Georgia: 3.3<br>Zambia: 5.0	2, 3, 4
Age structure (in bins of 5 years)	Brazil: [0.072, 0.078, 0.090, 0.089, 0.090, 0.090, 0.083, 0.073, 0.068, 0.062, 0.053, 0.043, 0.034, 0.025, 0.020, 0.013, 0.009, 0.004, 0.002, 0.001]<br>Georgia: [0.013, 0.057, 0.071, 0.064, 0.055, 0.058, 0.065, 0.073, 0.070, 0.065, 0.063, 0.062, 0.069, 0.064, 0.053, 0.039, 0.024, 0.025, 0.006, 0.006]<br>Zambia: [0.161, 0.165, 0.157, 0.101, 0.083, 0.068, 0.057, 0.051, 0.042, 0.030, 0.024, 0.015, 0.016, 0.009, 0.008, 0.005, 0.006, 0.002, 0.000, 0.000]	2, 3, 4
Minimum prime adult age	20 years	Assumed
Proportion of women	Brazil: 51%<br>Georgia: 52%<br>Zambia: 51%	5, 6, 7
Minimum working age	15 years	5, 6, 7
Employment rate	Brazil: 73% (male), 53% (female)<br>Georgia: 77% (male), 82% (female)<br>Zambia: 39% (male), 23% (female)	5, 6, 7
Formal employment rate	Brazil: 90% (male), 90% (female)<br>Georgia: 64% (male), 74% (female)<br>Zambia: 36% (male), 24% (female)	5, 6, 7
Schooling rate	Brazil: 97% (primary), 83% (secondary)<br>Georgia: 98% (primary), 95% (secondary)<br>Zambia: 79% (primary), 40% (secondary)	2, 3, 8
School gender parity	Brazil: 0.97 (primary), 0.98 (secondary)<br>Georgia: 1.00 (primary and secondary)<br>Zambia: 1.00 (primary), 0.90 (secondary)	2, 3, 8

Religious gathering participation rate	Brazil: 41%<br>Georgia: 13%<br>Zambia: 70%	9
Mean employment contacts (formal)	20	Assumed
Mean employment contacts (informal)	5	Assumed
Mean class size	Brazil: 20 (primary), 26 (secondary)<br>Georgia: 20 (primary and secondary)<br>Zambia: 37 (primary and secondary)	1, 10, 11
Mean school size	Brazil: 500 (primary), 400 (secondary) (assumed)<br>Georgia: 135 (primary and secondary)<br>Zambia: 700 (primary and secondary) (assumed)	5
Student/teacher ratio	Brazil: 20 (primary), 17 (secondary)<br>Georgia: 8 (primary and secondary)<br>Zambia: 42 (primary and secondary)	2, 12
Mean religious gathering size (standard dev.)	Brazil: 200 (100)<br>Georgia: 200 (100)<br>Zambia: 500 (100)	Assumed
Mean random contacts in religious gathering per person	10	Assumed
Mean random community contacts per day	10	Assumed

References for S1 Table.

1. Han AX, Hannay E, Carmona S, Rodriguez B, Nichols BE, Russell CA. Estimating the potential impact and diagnostic requirements for SARS-CoV-2 test-and-treat programs. Nat Commun. 2023 Dec 2; 14(1):7981.
2. Zambia Statistics Agency, Zambia Demographic and Health Survey 2018 (2018) (available at https://www.zamstats.gov.zm/).
3. Brazilian Institute of Geography and Statistics, IBGE | 2010 Census (2010) (available at https://censo2010.ibge.gov.br/).
4. National Statistics Office of Georgia, Census - მთავარი (2014) (available at https://www.geostat.ge/).
5. National Statistics Office of Georgia, საქართველოს სტატისტიკის ეროვნული სამსახური (2022) (available at https://www.geostat.ge/ka).

6. Zambia Statistics Agency, 2019 Labour Force Survey Report (2019) (available at https://www.zamstats.gov.zm/index.php/publications/category/7-labour).
7. Brazilian Institute of Geography and Statistics, Pesquisa Nacional por Amostra de Domicílios Contínua Trimestral - PNADC/T (2022) (available at https://sidra.ibge.gov.br/pesquisa/pnadct/tabelas).
8. UNESCO Institute for Statistics, Georgia | UNESCO UIS (2022) (available at http://uis.unesco.org/country/ge).
9. Pew Research Center, The Age Gap in Religion Around the World (2018) (available at https://www.pewresearch.org/religion/2018/06/13/the-age-gap-in-religion-around-the-world/).
10. OECD, Education at a Glance 2021 : OECD Indicators | Education at a Glance | OECD iLibrary (2021) (available at https://www.oecd-ilibrary.org/education/education-at-a-glance-2021_b35a14e5-en).
11. Ministry of Education and Science of Georgia, Teach & Learn With Georgia (2022) (available at http://www.tlg.gov.ge/content.php?id=643&lang=eng).
12. The World Bank, World Bank Open Data | Data (2022) (available at https://data.worldbank.org/).
