# Supplementary material for: Cost-effectiveness of SARS-CoV-2 self-testing at routine gatherings to minimize community-level infections in lower-middle income countries: A mathematical modeling study
Source: PLoS One. 2024 Oct 4;19(10):e0311198. doi: 10.1371/journal.pone.0311198 (PMC11451991; doi:10.1371/journal.pone.0311198)
Supplement: S2 Table — (PDF) [file pone.0311198.s002.pdf]

S2 Table. SARS-CoV-2 transmission related parameters used in the PATAT model [1].

Parameter	Values/Distribution	Reference
SARS-CoV-2 transmission related parameters		
Age-structured relative susceptibility (in bins of 5 years)	[0.34, 0.34, 0.67, 0.67, 1.00, 1.00, 1.00, 1.00, 1.00, 1.00, 1.00, 1.00, 1.00, 1.00, 1.24, 1.24, 1.47, 1.47, 1.47, 1.47]	2, 3
Age-structured probability of becoming symptomatic (in bins of 5 years)	[0.50, 0.50, 0.55, 0.55, 0.60, 0.60, 0.65, 0.65, 0.70, 0.70, 0.75, 0.75, 0.80, 0.80, 0.85, 0.85, 0.90, 0.90, 0.90, 0.90]	4, 5
Age-structured probability of developing severe disease (in bins of 5 years)	[0.00050, 0.00050, 0.00165, 0.00165, 0.00720, 0.00720, 0.02080, 0.02080, 0.03430, 0.03430, 0.07650, 0.07650, 0.13280, 0.13280, 0.20655, 0.20655, 0.24570, 0.24570, 0.24570, 0.24570]	4, 5
Age-structured probability of death (in bins of 5 years) (assuming 7% of severe infections lead to mortality)	[0.000035, 0.000035, 0.0001155, 0.0001155, 0.000504, 0.000504, 0.001456, 0.001456, 0.002401, 0.002401, 0.005355, 0.005355, 0.009296, 0.009296, 0.0144585, 0.0144585, 0.017199, 0.017199, 0.017199, 0.017199]	6
Latent period (days)	Omicron BA.1: Lognormal (4.0, 1.3)	3, 7, 8, 9
Pre-symptomatic period (days)	Omicron BA.1: Lognormal (1.8, 1.7)	3, 7, 8, 9
Period between symptom onset and severe disease (days)	Lognormal (6.6., 4.9)	7
Period between severe disease and death (days)	Lognormal (8.6, 6.7)	7
Recovery period for symptomatic agents with mild disease (days)	Omicron BA.1: Lognormal (5.35, 0.37*)	9, 10
Recovery period for asymptomatic agents (days)	Omicron BA.1: Lognormal (5.35, 0.37*)	9, 10
Recovery period for agents with severe disease (days)	Omicron BA.1: Lognormal (18.1, 6.3)	4
Peak Ct values	Omicron BA.1: Normal; Mean = 23.3, Standard dev. = 0.58*	9
Cross-immunity to variant virus after infection by extant virus	Omicron BA.1: 20%	11, 12
Severity (chance of hospitalization) of variant relative to extant virus	Omicron BA.1: 40%	13

*Standard deviation values inferred from 95% confidence interval computed in reference.

References for S2 Table.

1. Han AX, Hannay E, Carmona S, Rodriguez B, Nichols BE, Russell CA. Estimating the potential impact and diagnostic requirements for SARS-CoV-2 test-and-treat programs. Nat Commun. 2023 Dec 2; 14(1):7981.
2. J. Zhang, M. Litvinova, Y. Liang, Y. Wang, W. Wang, S. Zhao, Q. Wu, S. Merler, C. Viboud, A. Vespignani, M. Ajelli, H. Yu, Changes in contact patterns shape the dynamics of the COVID-19 outbreak in China. Science (1979) 368, 1481–1486 (2020).
3. C. C. Kerr, R. M. Stuart, D. Mistry, R. G. Abeysuriya, K. Rosenfeld, G. R. Hart, R. C. Núñez, J. A. Cohen, P. Selvaraj, B. Hagedorn, L. George, M. Jastrzębski, A. S. Izzo, G. Fowler, A. Palmer, D. Delport, N. Scott, S. L. Kelly, C. S. Bennette, B. G. Wagner, S. T. Chang, A. P. Oron, E. A. Wenger, J. Panovska-Griffiths, M. Famulare, D. J. Klein, Covasim: An agent-based model of COVID-19 dynamics and interventions. PLoS Comput Biol 17, e1009149-(2021).
4. R. Verity, L. C. Okell, I. Dorigatti, P. Winskill, C. Whittaker, N. Imai, G. Cuomo-Dannenburg, H. Thompson, P. G. T. Walker, H. Fu, A. Dighe, J. T. Griffin, M. Baguelin, S. Bhatia, A. Boonyasiri, A. Cori, Z. Cucunubá, R. FitzJohn, K. Gaythorpe, W. Green, A. Hamlet, W. Hinsley, D. Laydon, G. Nedjati-Gilani, S. Riley, S. van Elsland, E. Volz, H. Wang, Y. Wang, X. Xi, C. A. Donnelly, A. C. Ghani, N. M. Ferguson, Estimates of the severity of coronavirus disease 2019: a model-based analysis. Lancet Infect Dis 20, 669–677 (2020).
5. N. M. Ferguson, D. Laydon, G. Nedjati-Gilani, N. Imai, K. Ainslie, M. Baguelin, S. Bhatia, A. Boonyasiri, Z. Cucunubá, G. Cuomo-Dannenburg, A. Dighe, I. Dorigatti, H. Fu, K. Gaythorpe, W. Green, A. Hamlet, W. Hinsley, L. C. Okell, S. van Elsland, H. Thompson, R. Verity, E. Volz, H. Wang, Y. Wang, P. Gt Walker, C. Walters, P. Winskill, C. Whittaker, C. A. Donnelly, S. Riley, A. C. Ghani, Report 9: Impact of non-pharmaceutical interventions (NPIs) to reduce COVID-19 mortality and healthcare demand. (2020), doi:10.25561/77482.
6. Portmann L, de Kraker MEA, Fröhlich G, Thiabaud, A., Roelens, M., Schreiber, P. W., et al. Hospital Outcomes of Community-Acquired SARS-CoV-2 Omicron Variant Infection Compared With Influenza Infection in Switzerland. JAMA Netw Open. 2023;6(2):e2255599. Published 2023 Feb 1. doi:10.1001/jamanetworkopen.2022.55599
7. N. M. Linton, T. Kobayashi, Y. Yang, K. Hayashi, A. R. Akhmetzhanov, S. M. Jung, B. Yuan, R. Kinoshita, H. Nishiura, Incubation Period and Other Epidemiological Characteristics of 2019 Novel Coronavirus Infections with Right Truncation: A Statistical Analysis of Publicly Available Case Data. Journal of Clinical Medicine 2020, Vol. 9, Page 538 9, 538 (2020).
8. M. Kang, H. Xin, J. Yuan, S. Taslim Ali, Z. Liang, J. Zhang, T. Hu, E. H. Y Lau, Y. Zhang, M. Zhang, B. J. Cowling, Y. Li, P. Wu, P. Health, L. Ka Shing, Transmission dynamics and epidemiological characteristics of Delta variant infections in China. medRxiv , 2021.08.12.21261991 (2021).
9. J. A. Hay, S. M. Kissler, J. R. Fauver, C. Mack, C. G. Tai, R. M. Samant, S. Connelly, D. J. et al. Quantifying the impact of immune history and variant on SARS-CoV-2 viral kinetics and infection rebound: A retrospective cohort study. Elife. 2022;11:e81849. Published 2022 Nov 16. doi:10.7554/eLife.81849
10. R. Wölfel, V. M. Corman, W. Guggemos, M. Seilmaier, S. Zange, M. A. Müller, D. Niemeyer, T. C. Jones, P. Vollmar, C. Rothe, M. Hoelscher, T. Bleicker, S. Brünink, J. Schneider, R. Ehmann, K. Zwirglmaier, C. Drosten, C. Wendtner, Virological assessment of hospitalized patients with COVID2019. Nature 2020 581:7809 581, 465–469 (2020).
11. K. B. Pouwels, E. Pritchard, P. C. Matthews, N. Stoesser, D. W. Eyre, K.-D. Vihta, T. House, J. Hay, J. I. Bell, J. N. Newton, J. Farrar, D. Crook, D. Cook, E. Rourke, R. Studley, T. Peto, I. Diamond, A. S. Walker, the C.-19 I. S. Team, Impact of Delta on viral burden and vaccine

effectiveness against new SARS-CoV-2 infections in the UK. medRxiv , 2021.08.18.21262237 (2021).
12. Imperial College London, Report 49 - Growth, population distribution and immune escape of Omicron in England | Faculty of Medicine | Imperial College London (2021) (available at https://www.imperial.ac.uk/mrc-global-infectious-disease-analysis/covid-19/report-49-Omicron/).
13. Imperial College London, Report 50 - Hospitalisation risk for Omicron cases in England | Faculty of Medicine | Imperial College London (2022) (available at https://www.imperial.ac.uk/mrc-globalinfectious-disease-analysis/covid-19/report-50-severity-omicron/).
