# Supplementary material for: Cost-effectiveness of SARS-CoV-2 self-testing at routine gatherings to minimize community-level infections in lower-middle income countries: A mathematical modeling study
Source: PLoS One. 2024 Oct 4;19(10):e0311198. doi: 10.1371/journal.pone.0311198 (PMC11451991; doi:10.1371/journal.pone.0311198)
Supplement: S3 Table. Testing parameters used in the PATAT model — (PDF) [file pone.0311198.s003.pdf]

S3 Table. Testing parameters used in the PATAT model [1].

Parameter	Values/Distribution	Reference
Testing parameters		
Delay in visiting healthcare facility for symptomatic testing (days)	Lognormal; Mean = 1.0, Standard dev. = 0.5	Assumed
Ag-RDT specificity	0.989	2
Ag-RDT sensitivity by Ct value	Ct $<$ 20: 0.965<br>Ct $<$ 25: 0.958<br>Ct $\geq$ 25: 0.507<br>Ct $\geq$ 30: 0.209	2

References for S3 Table.

1. Han AX, Hannay E, Carmona S, Rodriguez B, Nichols BE, Russell CA. Estimating the potential impact and diagnostic requirements for SARS-CoV-2 test-and-treat programs. Nat Commun. 2023 Dec 2; 14(1):7981.
2. Brümmer LE, Katzenschlager S, Gaeddert M, Erdmann, C., Schmitz, S., Bota, M., et al. Accuracy of novel antigen rapid diagnostics for SARS-CoV-2: A living systematic review and meta-analysis [published correction appears in PLoS Med. 2021 Oct 13;18(10):e1003825]. PLoS Med. 2021;18(8):e1003735. Published 2021 Aug 12. doi:10.1371/journal.pmed.1003735
