# Supplementary material for: Cost-effectiveness of SARS-CoV-2 self-testing at routine gatherings to minimize community-level infections in lower-middle income countries: A mathematical modeling study
Source: PLoS One. 2024 Oct 4;19(10):e0311198. doi: 10.1371/journal.pone.0311198 (PMC11451991; doi:10.1371/journal.pone.0311198)
Supplement: S4 Table — (PDF) [file pone.0311198.s004.pdf]

S4 Table. Isolation and quarantine parameters used in the PATAT model [1].

Parameter	Values/Distribution	Reference
Isolation and quarantine parameters		
Isolation period	7 days	Assumed
Quarantine period	7 days	Assumed
Reduction in contact rates under isolation/quarantine (in order of households, schools, workplaces, religious gathering and random community)	[10%, 100%, 100%, 100%, 100%]	Assumed

References for S4 Table.

1. Han AX, Hannay E, Carmona S, Rodriguez B, Nichols BE, Russell CA. Estimating the potential impact and diagnostic requirements for SARS-CoV-2 test-and-treat programs. Nat Commun. 2023 Dec 2; 14(1):7981.
