# Supplementary material for: Cost-effectiveness of SARS-CoV-2 self-testing at routine gatherings to minimize community-level infections in lower-middle income countries: A mathematical modeling study
Source: PLoS One. 2024 Oct 4;19(10):e0311198. doi: 10.1371/journal.pone.0311198 (PMC11451991; doi:10.1371/journal.pone.0311198)
Supplement: S5 Table — (PDF) [file pone.0311198.s005.pdf]

S5 Table. CHEERS checklist [1].

Topic	*No.*	*Item*	*Location where item is reported*
**Title**	1	Identify the study as an economic evaluation and specify the interventions being compared.	Title
**Abstract**	2	Provide a structured summary that highlights context, key methods, results, and alternative analyses.	Abstract
Introduction			
**Background and objectives**	3	Give the context for the study, the study question, and its practical relevance for decision making in policy or practice.	Introduction, start at line 54 & 97
Methods			
**Health economic analysis plan**	4	Indicate whether a health economic analysis plan was developed and where available.	Materials and methods, start at line 180
**Study population**	5	Describe characteristics of the study population (such as age range, demographics, socioeconomic, or clinical characteristics).	Materials and methods, start at line 124
**Setting and location**	6	Provide relevant contextual information that may influence findings.	Materials and methods, start at line 124
**Comparators**	7	Describe the interventions or strategies being compared and why chosen.	Materials and methods, start at line 143
**Perspective**	8	State the perspective(s) adopted by the study and why chosen.	Materials and methods, line 184
**Time horizon**	9	State the time horizon for the study and why appropriate.	Materials and methods, line 257
**Discount rate**	10	Report the discount rate(s) and reason chosen.	Materials and methods, line 183

Topic	*No.*	*Item*	*Location where item is reported*
**Selection of outcomes**	11	Describe what outcomes were used as the measure(s) of benefit(s) and harm(s).	Materials and methods, line 187
**Measurement of outcomes**	12	Describe how outcomes used to capture benefit(s) and harm(s) were measured.	Materials and methods, line 187
**Valuation of outcomes**	13	Describe the population and methods used to measure and value outcomes.	Materials and methods, start at line 124
**Measurement and valuation of resources and costs**	14	Describe how costs were valued.	Materials and methods, start at line 180
**Currency, price date, and conversion**	15	Report the dates of the estimated resource quantities and unit costs, plus the currency and year of conversion.	Materials and methods, start at line 180
**Rationale and description of model**	16	If modelling is used, describe in detail and why used. Report if the model is publicly available and where it can be accessed.	Materials and methods, start at line 108
**Analytics and assumptions**	17	Describe any methods for analysing or statistically transforming data, any extrapolation methods, and approaches for validating any model used.	Materials and methods, start at line 108
**Characterising heterogeneity**	18	Describe any methods used for estimating how the results of the study vary for subgroups.	Materials and methods, starting at line 108
**Characterising distributional effects**	19	Describe how impacts are distributed across different individuals or adjustments made to reflect priority populations.	Materials and methods, starting at line 108
**Characterising uncertainty**	20	Describe methods to characterise any sources of uncertainty in the analysis.	Materials and methods, starting at line 108
**Approach to engagement with patients and others affected by the study**	21	Describe any approaches to engage patients or service recipients, the general public, communities, or stakeholders (such as clinicians or payers) in the design of the study.	Not applicable
Results			

Topic	*No.*	*Item*	*Location where item is reported*
**Study parameters**	22	Report all analytic inputs (such as values, ranges, references) including uncertainty or distributional assumptions.	Table 1 and 2
**Summary of main results**	23	Report the mean values for the main categories of costs and outcomes of interest and summarise them in the most appropriate overall measure.	Table 1, 2, and 3
**Effect of uncertainty**	24	Describe how uncertainty about analytic judgments, inputs, or projections affect findings. Report the effect of choice of discount rate and time horizon, if applicable.	Materials and methods, starting at line 108
**Effect of engagement with patients and others affected by the study**	25	Report on any difference patient/service recipient, general public, community, or stakeholder involvement made to the approach or findings of the study	Not applicable
**Discussion**			
**Study findings, limitations, generalisability, and current knowledge**	26	Report key findings, limitations, ethical or equity considerations not captured, and how these could affect patients, policy, or practice.	Results and discussion, starting at line 202
**Other relevant information**			
**Source of funding**	27	Describe how the study was funded and any role of the funder in the identification, design, conduct, and reporting of the analysis	Line 498
**Conflicts of interest**	28	Report authors conflicts of interest according to journal or International Committee of Medical Journal Editors requirements.	Line 487

References for S5 Table.

1. Husereau D, Drummond M, Augustovski F, et al. Consolidated Health Economic Evaluation Reporting Standards 2022 (CHEERS 2022) Explanation and Elaboration: A Report of the ISPOR CHEERS II Good Practices Task Force. Value Health 2022;25. doi:10.1016/j.jval.2021.10.008
